# Refining the Behavioral Phenotype of Angelman Syndrome: Examining Differences in Motivation for Social Contact Between Genetic Subgroups

**DOI:** 10.3389/fnbeh.2021.618271

**Published:** 2021-02-16

**Authors:** Mary Heald, Dawn Adams, Emily Walls, Christopher Oliver

**Affiliations:** ^1^Cerebra Centre for Neurodevelopmental Disorders, School of Psychology, University of Birmingham, Birmingham, United Kingdom; ^2^Autism Centre of Excellence, Griffith University, Mount Gravatt, QLD, Australia

**Keywords:** Angelman syndrome, genomic imprinting, kinship theory, social behavior, operant learning, behavioral phenotype

## Abstract

Angelman syndrome (AS) is caused by loss of information from the 15q11.2-13 region on the maternal chromosome with striking phenotypic difference from Prader–Willi syndrome in which information is lost from the same region on the paternal chromosome. Motivation for social contact and sensory seeking behaviors are often noted as characteristics of the phenotype of AS and it has been argued that the strong drive for social contact supports a kinship theory interpretation of genomic imprinting. In this study we developed an experimental paradigm for quantifying the motivation for social contact in AS and examined differences across the genetic subtypes that cause AS [deletion, imprinting centre defect (ICD), uniparental disomy and UBE3A mutation]. Using single case experimental designs we examined the rate of acquisition of behavioral responses using operant learning paradigms for 21 children with AS whilst systematically varying the nature of social and sensory reinforcement. Variability in rates of acquisition was influenced by the nature of rewarding stimuli. Across the total sample both sensory stimuli and social contact could increase the rate of rewarded behavior with difference between children in the most effective reward. A striking difference in the rewarding properties of social contact across genetic subtypes was evidenced by non-deletion genetic causes of AS showing significantly higher rates of responding than the deletion cause in the social reinforcement paradigm. The results indicate that reinforcer assessment can beneficially inform behavioral interventions and that within syndrome variability in the behavioral phenotype of AS is likely driven by genetic difference. The non-deletion cause of AS, and particularly the ICD group, may be the optimal group for further study of genomic imprinting.

## Introduction

Angelman syndrome (AS) is a rare genetic disorder caused by missing information within the 15q11.2-13 region on the maternal chromosome, and prevalent in approximately 1 in 10,000 live births (Buckley et al., 1998). Four genetic mechanisms cause AS, each involving disruption to the UBE3A gene (Jiang et al., 1998): *de novo* deletion (approximately 70% of cases; Knoll et al., 1989), uniparental disomy (UPD; 2%; Engel, 1993; Prasad and Wagstaff, 1997), UBE3A mutation (2–8%; Kishino et al., 1997) and an imprinting center defect (ICD; 2.5%; Bürger et al., 1997). A subset of cases are clinically diagnosed with no identifiable genetic cause (Peters et al., 2004). AS is associated with a range of physical characteristics including seizures, atypical facial features (Williams et al., 2006), abnormal EEG (Boyd et al., 1988), and ataxic gate (Dan and Chéron, 2004).

The cognitive, social and behavioral phenotype of AS is well delineated. Severe to profound intellectual disability is typically evident, with deficits across adaptive behaviors and cognitive domains (Peters et al., 2004; Gentile et al., 2010). Strengths in socialization (Peters et al., 2004) are evident but there is greater impairment of learning and attention (Walz and Benson, 2002; Jiang et al., 2010). Genotype-phenotype correlations are described (Gentile et al., 2010), with a *de novo* deletion associated with greater impairments across all areas of cognition and behavior compared to ICD, UPD, and UBE3A mutation. Broad communication impairments are typical (Clayton-Smith and Laan, 2003), with notable deficits in expressive compared to receptive language, and the vast majority of children and adults are non-verbal with limited alternative communication skills (Jolleff and Ryan, 1993; Penner et al., 1993; Calculator and Black, 2010; Pearson et al., 2019). Notably, AS is characterized by frequent laughing and smiling (Horsler and Oliver, 2006a), enhanced sociability (Mount et al., 2011), a high prevalence of aggressive behavior (Arron et al., 2011), heightened impulsivity (Oliver et al., 2011), short attention span (Walz and Benson, 2002), and sleep difficulties (Pelc et al., 2008). AS is also associated with unusual responses to sensory stimuli and experiences (Walz and Baranek, 2006; Peters et al., 2012) with sensory *seeking* behaviors, as opposed to hyper or hypo-sensitivity, characterizing the sensory processing profile (Heald et al., 2020). Sensory seeking behavior has not been explored in learning paradigms or with regard to genetic difference in AS. Study of these two areas will inform interventions and further delineate genotype-phenotype associations.

Of particular interest are the distinctive social behaviors, in part due to the potential for exploring the social and behavioral manifestation of genomic imprinting in humans. Whilst many genes have the same effect regardless of the parent of origin (maternal or paternal chromosome), a small number of genes are expressed differently depending on whether they are inherited from the mother or father, a phenomenon known as genomic imprinting. Angelman and the “sister disorder” Prader–Willi syndrome, highlight the effects of genomic imprinting as they are caused by missing information on the same 15q11-13 region of the maternal and paternal chromosomes, respectively, with markedly contrasting phenotypes. Kinship theory (Haig and Wharton, 2003; Brown and Consedine, 2004) proposes that for genes where the parent of origin is important, as in AS, paternal genes may favor behavioral expression which increases the cost to the mother of the offspring and vice versa for maternal genes. It has been proposed that the striking social behaviors observed in AS support kinship theory due to their effectiveness in securing social resources. For example, the heightened laughing and smiling in AS demonstrably elicits greater social resources from adults in competitive settings compared to the same behavior shown by children with intellectual disability of heterogeneous cause (Oliver et al., 2007). Kinship theory is further supported by mouse models of AS, with increased ultrasonic vocalizations in maternal deletion mice in the presence of maternal bedding compared to wild-type mice (Jiang et al., 2010). Whilst compelling, the kinship theory of genomic imprinting in AS has been relatively unexplored, as have possible differences across genetic subtypes. Study of the latter might identify optimal genetic subgroups for further exploration of genomic imprinting. Additionally, it is important to develop an experimental paradigm that can quantify the drive for social contact.

Understanding the profile of social behavior in AS also has the potential to enhance skill acquisition and help decrease phenotypic behavior, such as aggression, by identifying rewarding properties of social and sensory stimuli and hence likely motivation for learning. Comparatively slow rates of acquisition are frequently noted in applications of standardized interventions with children and adults with AS (Summers and Szatmari, 2009; Summers, 2012; Heald et al., 2013), a finding supported by parental report (Calculator, 2002). It is possible that this underpins the paucity of literature on intervention in AS and few studies have examined the efficacy of standardized behavioral interventions designed for the intellectual disability population to address difficulties reported within the syndrome, including compromised acquisition of adaptive behavior (Gentile et al., 2010), aggression (Arron et al., 2011), sleep problems (Didden et al., 2004), and very strong motivation for social contact (Oliver et al., 2011). There is a pressing need to develop and explore methodologies for children and adults with AS that support effective intervention and, crucially, provide a metric of change.

Given the hypotheses regarding social contact in AS derived from kinship theory and the importance of identifying reinforcer efficacy for the purpose of changing behavior, there is a robust rationale for examining the potential role of social behaviors, sensory stimuli, and reward preference in learning.

The main aim of the current study was to examine the rewarding effect of social and sensory stimuli on the speed of acquisition of target behaviors in children with AS across different genetic causes of AS. A secondary aim was to determine the components of social stimuli that enhance acquisition in learning paradigms. Fulfilling these aims will: (1) identify whether genetic subgroups in AS find social contact more rewarding and hence, might be optimal groups for further study of kinship theory and social behavior resulting from genomic imprinting and (2) inform intervention strategies with regard to reinforcer identification.

## Materials and Methods

### Participants

Participants were 21 children with AS aged between 4 and 15 years.^1^ Participants were recruited through the large database of families held at the Cerebra Centre for Neurodevelopmental Disorders and the UK Angelman syndrome support group: the Angelman Syndrome Support Education and Research Trust (ASSERT). Inclusion criteria included a genetic diagnosis of AS from a relevant professional (e.g., a clinical geneticist). All genetic subtypes of AS were recruited including deletion (*n* = 14),^2^ UBE3A mutation (*n* = 1), ICD (*n* = 5), and UPD (*n* = 1). Due to low numbers of participants across genetic subtypes of AS, two groups were created: children with and without a deletion. A similar analysis strategy has been employed in the AS literature to examine genotype × phenotype correlations in adaptive behavior, cognition, and autism spectrum disorder (ASD) characteristics (Mertz et al., 2014). Table 1 shows the participant demographics across deletion and non-deletion groups.

**TABLE 1:** Mean age in years (standard deviation), gender, adaptive behavior skills, Autism Spectrum characteristics, and aggression in children with Angelman syndrome divided by genetic subtype: “deletion” (Class I and II) and “non deletion” (imprinting center defect, UBE3A mutation and uniparental disomy).

		**Deletion**	**Non deletion**	**F/X^2^**	**df**	***p***
*N*		14	7			
Age (years)	Mean (SD)	12.07 (3.73)	8.14 (3.98)	4.96	1	0.04*
	Range	4–15	5–15			
Gender	% Male	50.0	28.6	0.88	1	0.64
Self help score^a^	Mean (SD)	4.14 (0.53)	6.29 (1.11)	36.54	1	<0.01**
Mobility^a^	% Mobile	79.6 (11)	100.0 (7)	1.75	1	0.52
Vision^a^	% Normal	93.9 (13)	100 (7)	0.53	1	1.00
Hearing^a^	% Normal	100.0 (14)	100 (7)	–	–	–
Speech^a^	% Verbal	0.0 (0)	0.0 (0)	–	–	–
SCQ^b^	Communication	11.02 (2.79)	4.41 (4.47)	−3.05	1	0.002**
	Social interaction	8.06 (2.68)	3.57 (1.40)	−3.08	1	0.002**
	Repetitive behavior	4.14 (1.79)	4.00 (1.53)	−0.38	1	0.74
	Total score	19.85 (4.29)	11.43 (4.50)	−3.07	1	0.001**
Aggression	% Presence (*n*)	71.4 (10)	100 (7)	2.47	1	0.26

*p < 0.05, **p < 0.01. ^*a*^Information obtained from the Wessex Scale (Kushlick et al., 1973). ^*b*^Information obtained from the Social Communication Questionnaire (SCQ; Rutter et al., 2003). ^*c*^Information obtained from the Challenging Behavior Questionnaire (Hyman et al., 2002).

Comparisons of demographic characteristics were conducted across deletion and non-deletion groups. Children with a deletion were significantly older, and evidenced a higher frequency of ASD characteristics and a lower level of self-help skills. The observed differences are consistent with previous research examining adaptive behavior and ASD across genetic subtypes (Gentile et al., 2010), including comparisons across deletional and non deletional subtypes of AS (Moncla et al., 1999). There was no significant difference in other behaviors including mobility, vision, hearing, speech, and presence of aggression.

### Measures

#### Demographic Questionnaire

Informants provide demographic characteristics including date of birth, gender, mobility, speech, and genetic diagnosis, including details about the genetic mechanism and professional diagnosing the syndrome.

#### The Wessex Behavior Scale

The Wessex Behavior Scale is a 15 item questionnaire originally part of a larger measure of behavior in individuals with an intellectual disability (Kushlick et al., 1973). The Wessex is a measure of adaptive behavior and covers self-help skills, mobility, vision, reading, writing, and continence. The Wessex produces a total score out of nine, with higher scores indicating a greater level of adaptive behavior. The measure has good inter-rater reliability (Kushlick et al., 1973; Palmer and Jenkins, 1982).

#### Social Communication Questionnaire

The Social Communication Questionnaire (SCQ) is a 40 item informant report questionnaire which assesses the presence of ASD characteristics (Rutter et al., 2003). It is composed of three subscales: *social interaction*, *communication*, and *stereotyped patterns of behaviors*. The SCQ is an ASD screening questionnaire, with higher scores indicating a greater presence of ASD characteristics. The SCQ has good internal consistency (Berument et al., 1999).

#### Challenging Behavior Questionnaire

The Challenging Behavior Questionnaire (CBQ) is an eight item informant report questionnaire which assesses the presence of aggression, self-injury, destruction and stereotyped behaviors (Hyman et al., 2002). The reliability coefficients of the CBQ range from 0.61 to 0.89, indicating good inter-rater reliability (Hyman et al., 2002).

### Procedure

Participants were visited at school or home to complete the experimental observations. The experimental visit was conducted in an empty room, where possible free from distracting objects and preferred items. Where possible, only the researchers and the participant were present. For six participants, a teacher was present for the experimental observations but did not interact with the child.

#### Engagement Preference Assessment

In order to select preferred and non-preferred stimuli for the sensory reinforcement learning paradigm, an “engagement preference assessment” (EPA) presents items individually to children and records the interaction with the stimuli. Although the “Forced-Choice Assessment” (Fisher et al., 1992) is the most widely used assessment of preference, as both methods have been validated with individuals with an intellectual disability (Hagopian et al., 2001; Keen and Pennell, 2010) the EPA, which gives additional information about satiation and motivation for adult attention, was chosen.

During the preference assessment, 12 stimuli, toys, and other items, were presented. The order of stimulus presentation was counterbalanced across participants. The researcher presented participants with each stimulus and directed their attention toward it, e.g., “look, <child’s name>.” No further social interaction was given. Participants were given 2 min of access to each toy. Although this is comparatively shorter than previous studies using this procedure, during a pilot study participants became distressed due to the withdrawal of adult interaction.

The total time the participant engaged with each stimulus was recorded. From this, the highest and lowest preference items were determined using the longest and shortest time touching the stimulus. If there was no difference in the total engagement times across stimuli, a forced choice preference assessment was conducted (Fisher et al., 1992). In the forced choice preference assessment, items with which the child engaged for an equal amount of time were presented concurrently, and the item chosen by the participant was taken as preferred. Two items were presented at a time, and items were presented in every possible combination. A forced choice preference assessment was employed with two participants.

#### Reinforcement Assessments

Both sensory and social reinforcement and their effect on speed of acquisition of target behaviors were assessed individually. Target behaviors were different across participants and based on ability. Specifically, it was ensured that target behaviors were non-demanding in order to avoid the rate of behavior being affected by a propensity to escape from task demands (Strachan et al., 2009). Examples of behaviors that were chosen include touching a certain object or executing a certain action. The order of reinforcement assessments (social/sensory) was counterbalanced among participants.

There were two experimental sessions for both the sensory and social reinforcement assessments with single case experimental designs employed. Sessions were of a withdrawal ABACA design for the sensory reinforcement assessment, and ABACADAEAFA for the social reinforcement assessment. Both took the form of contingent reinforcement with an FR1 schedule. Each condition lasted for 2 min. In the withdrawal (ABA) design, A (withdrawal/withholding of reinforcer), is alternated with experimental conditions where a specific reinforcer is presented (B, C, D, etc.). The order of experimental conditions was counterbalanced across sessions. Before each condition, participants received a primer in order to indicate the reward received contingent on target behaviors. In the primer, the researcher prompted the target behavior using three point teaching procedure: verbal prompt, physical prompt, and hand over hand prompt. Contingent on this target behavior, participants were given the specified reinforcement depending on the experimental condition. All participants then progressed to the experimental condition. In order to overcome possible effects of memory and attention, a verbal prompt was given at 1 min. During each the researcher sat in proximity to the participant but did not engage in any interaction (verbal, physical, eye contact, and laughing/smiling) unless prompting target behavior or as part of the reinforcement.

#### Sensory Reinforcement Assessment

There were two experimental sessions with an ABACA withdrawal design. The order of conditions was counterbalanced across sessions and across participants. In the control/withdrawal condition (A), participants received no reinforcement upon completion of the target behavior. In the preferred item condition (B), participants were given 5 s of access to the preferred tangible (identified in the preference assessment) for displaying the target behavior. In the least preferred item condition (C) participants were given 5 s of access to their least preferred tangible (identified in the preference assessment) contingent on the target behavior being displayed. Throughout the conditions the least and most preferred items were out of sight from participants.

#### Social Reinforcement Assessment

There were two experimental sessions with a withdrawal ABACADAEAFA design. The order of conditions was counterbalanced across sessions and across participants. In the control/withdrawal condition (A) participants received no form of reinforcement upon completion of the target behavior. In the social interaction condition (B), participants received full social interaction if the target behavior was performed (physical contact, verbal interaction, laughing and smiling, and eye contact). In condition C (restricted eye contact), participants received full social interaction but without eye contact. In condition D (restricted physical interaction), participants received social interaction without any physical contact. In condition E (restricted verbal interaction), participants received social interaction with no verbal interaction contingent on the target behavior. In condition F (no laughing or smiling) participants received social interaction contingent on the target behavior, without the researcher laughing/smiling.

### Analysis

Experimental visits were video recorded in order to allow for the coding of participants’ behavior during the assessments.

#### Reinforcer Assessments

The mean (across repeats of conditions) frequency of target behaviors for social, sensory, and control conditions was calculated for each participant. In order to establish that the learning paradigm was generally effective, i.e., that children would show higher rates of target behaviors in the presence of a reward, the frequency of target behaviors in reinforcement conditions was compared to control conditions using Wilcoxon signed ranks tests. In order to address the hypothesis that target behaviors would vary according to the *type* of social interaction given as a reward, Friedman tests were employed to compare the frequency of target behaviors across specific conditions of social interaction (eye contact, physical interaction, verbal interaction, laughing and smiling, and full social interaction) with Wilcoxon signed ranks test *Post Hocs*. To establish the integrity of the preference assessment, comparisons across specific conditions of sensory reinforcement (high preference and low preference) were conducted using Wilcoxon signed ranks tests.

In order to compare the effect of reinforcers across different sensory, social conditions, and control conditions, dominance statistics were calculated (d-statistic; Cliff, 1993). The d-statistic provides a measure of how much the distribution of the frequency of behaviors in one condition lies above the distribution of behaviors in a comparison condition. To calculate the d-statistic, data points from reinforcement conditions were compared to each datum point from control conditions. In order to produce datum points, the total number of target behaviors in each 30 s interval in 2 min conditions was calculated. Hence, each condition produced four datum points. Dominance matrices are used to calculate the d-statistic. For each cell, a value of +1 is given if the frequency of target behavior in the reinforcement condition is higher than the control condition, −1 if it is less than the control condition and 0 if there is no difference. From this the d-statistic can be calculated. In order to quantify whether a stimulus was reinforcing, an arbitrary cut-off of 0.5 was used. The proportion of participants that exceeded a d-statistic of 0.5 across each social and sensory condition was then calculated. In order to address the third aim of the study, to examine the comparative efficacy of social and sensory rewards as reinforcers, the number of participants exceeding a d-statistic cut-off of 0.5 across sensory and social rewards was examined, in addition to comparing the frequency of target behaviors across conditions using Wilcoxon signed ranks tests.

#### Genetic Subtype of Angelman Syndrome

To examine difference across genetic subtypes of AS (deletion vs. non deletion), the proportion of participants who were reinforced by a social and/or sensory reinforcement condition (d-statistic > 0.5) was calculated, and differences across groups calculated using Fishers exact test. As over 70% (5/7) of the sample of participants in the non-deletion group had AS cause by an ICD, the proportion of participants exceeding a d-statistic over 0.5 was also calculated for ICD alone.

## Results

**FIGURE 1:**
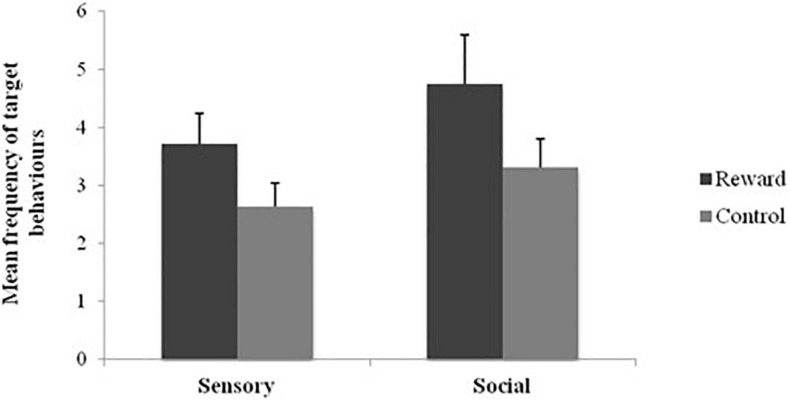
Mean frequency of target behaviors across social and sensory reinforcement and control conditions.

The first aim of the study was to examine the comparative efficacy of social and sensory stimuli as reinforcers. Overall, 38.1% of the total sample’s behaviors were reinforced by the presentation of sensory stimuli (indicated by a d-statistic exceeding 0.5), with target behaviors significantly higher compared to control conditions (*Z* = −3.13, *p* < 0.01, *r* = 0.68; see Figure 1). In comparison, 47.6% of participants were reinforced by social stimuli, with target behaviors significantly higher when given social rewards compared to control conditions (*Z* = −2.10, *p* = 0.03, *r* = 0.46; see Figure 1). There was no significant difference across the mean number of target behaviors across sensory and social reinforcement either across all sensory and social reinforcement conditions (*Z* = −1.18, *p* = 0.24, *r* = 0.26), or across the conditions with the highest frequency of target behaviors: high preference sensory stimuli and no physical social interaction conditions (*Z* = −0.06, *p* = 0.95, *r* = 0.01).

### Social Reinforcement Assessment

To address the second aim to examine the components of social contact which would provide stronger rewards, the frequency of target behaviors and associated d statistics across relevant conditions were compared. Table 2 shows the mean frequency of target behaviors and d-statistics across social reinforcement and control conditions.

**TABLE 2:** The mean frequency (standard deviation) of target behaviors, associated d-statistics and number of participants exceeding d-statistic cut-offs across social interaction conditions (full social interaction and full social interaction minus physical interaction, eye contact, laughing/smiling, verbal interaction).

	**Reward conditions**							
	**Full social interaction**	**No physical**	**No eye contact**	**No laughing**	**No verbal interaction**	**Control condition**	**X^2^**	***p***
Mean frequency of target behavior	5.07 (4.23)	5.40 (5.02)	4.77 (4.77)	4.24 (3.67)	4.26 (4.35)	2.63 (1.87)	6.26	0.23
Mean d-statistic	0.21	0.21	0.12	0.12	0.10	–	4.23	0.37
>0.5 (%)	19.0 (4)	33.3 (7)	19.0 (4)	9.5 (2)	14.3 (3)	–	–	–

The results show no significant differences in the mean frequency of target behaviors across social reinforcement and control conditions (*X*^2^ = 6.26, *p* = 0.23), or across social reinforcement conditions alone (*X*^2^ = 5.85, *p* = 0.21). There were no significant differences in mean d-statistics across social reinforcement conditions (*X*^2^ = 4.23, *p* = 0.37).

**FIGURE 2:**
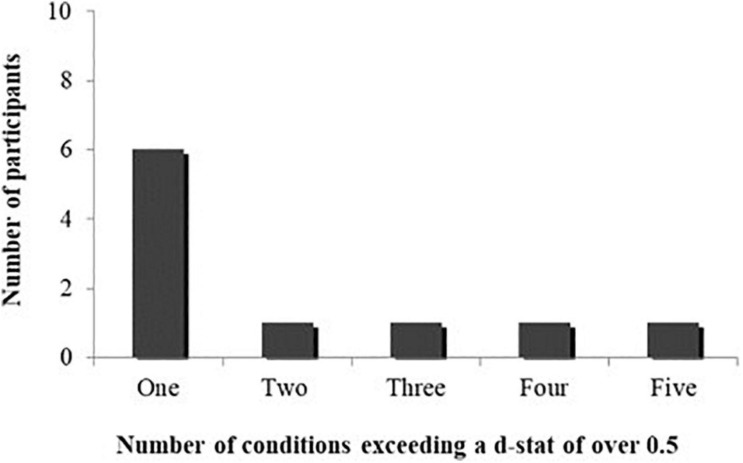
The number of social reinforcement conditions where participants exceed a cut off of 0.5 for the d-statistic.

Whilst no consistent reinforcer was found across conditions, further examination of the d-statistic revealed that for each participant, *specific* combinations or elements of components of social rewards functioned as reinforcers rather than all rewards equally. Figure 2 shows the number of participants who were reinforced by only one social condition (indicated by a d-statistic of over 0.5), two conditions and so on to a maximum of five social interaction conditions. The results show that 60% of participants reinforced by social stimuli were only reinforced by *one* social interaction condition.

### Reinforcement and Demographic Characteristics

#### Age and Adaptive Behavior

Having established the integrity of the learning paradigm, the relationship between adaptive behavior and age, adaptive behavior, and reinforcer efficacy was examined to determine possible influences on learning in person characteristics that differed across genetic subgroups. Spearman Rho correlations were conducted between the Wessex total self-help score, age, and the frequency of target behaviors across all conditions and each condition individually. The results show no significant association between self-help scores and target behaviors during sensory reinforcement conditions (*R* = 0.23, *p* = 0.31) or social reinforcement conditions (*R* = 0.26, *p* = 0.26). There was also no significant association between age and target behaviors during sensory reinforcement conditions (*R* = −0.11, *p* = 0.62) or social reinforcement conditions (*R* = −0.20, *p* = 0.38).

#### Genetic Subtype

The possible influence of genetic subtype on the strength of social or sensory reinforcers contrasts across groups were made. Table 3 shows the proportion of children receiving a d-statistic over 0.5 for any of the social and/or sensory reinforcement conditions across individuals with a deletion (*n* = 14) and without a deletion (*n* = 7). Fisher’s exact tests revealed no significant difference in the number of participants reinforced by sensory stimuli across genetic groups (*p* = 0.17). However, significantly more participants without a deletion were reinforced by social stimuli (*p* = 0.02).

**TABLE 3:** Proportion of participants reinforced by sensory and/or social stimuli, defined as a d-statistic over 0.5.

	**Social**	**Sensory**
Deletion (*n* = 14)	28.6 (4/14)	50.0 (7/14)
Non deletion (*n* = 7)	85.7 (6/7)	14.3 (1/7)
ICD (*n* = 5)	100.0 (5/5)	20.0 (1/5)

*Proportions are shown for participants with and without a deletion, and children with imprinting center defect (ICD).*

**FIGURE 3:**
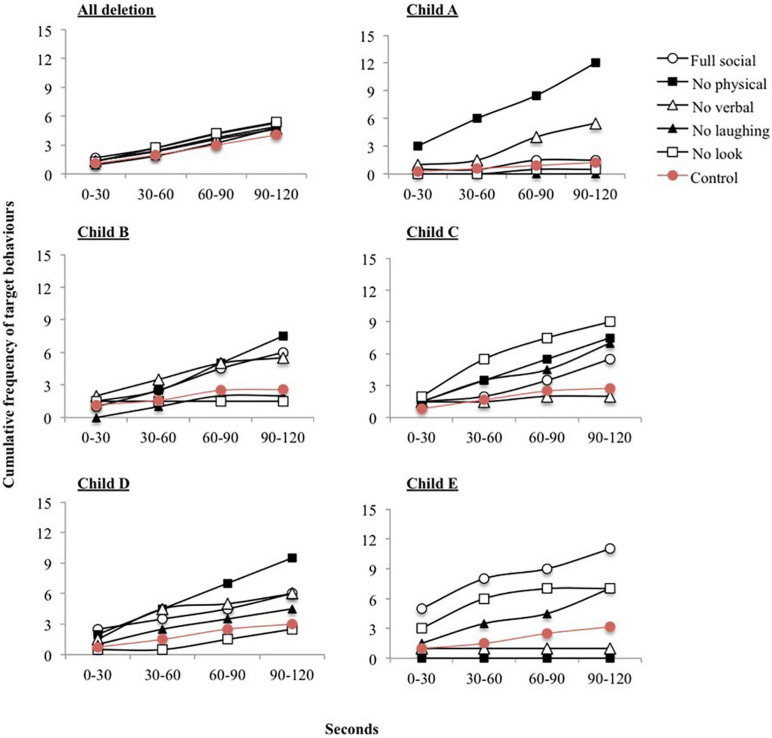
Cumulative frequency of target behaviors across each 30 second interval during social reinforcement conditions. Graphs are shown for all participants with a deletion and each participant with Imprinting Centre Defect (ICD) separately.

Table 3 also shows the number of participants with ICD who were reinforced by sensory/social stimuli. All children with ICD were reinforced by social stimuli. Fisher’s exact tests revealed no significant difference in the number of participants reinforced by sensory stimuli across ICD and deletion groups (*p* = 0.34). However, significantly more participants with ICD were reinforced by social stimuli (*p* = 0.01). Figure 3 shows the cumulative frequency graphs for all children with a deletion compared to all five individual children with ICD separately. Each individual graph for children with ICD shows higher levels of target behavior in one or more of the social conditions than children with a deletion.

## Discussion

The main aim of the current study was to examine the drive for social contact in children with AS by establishing the comparative efficacy of social interaction as a reward on the rate of acquisition in a learning paradigm in children with AS. This is the first study to establish the systematic evaluation of different reinforcers to increase the slow rate of learning frequently reported in AS. It is encouraging for practitioners that all children were reinforced by either sensory or social stimuli, as evidenced by higher rates of target behavior in the presence of the reinforcer. Across the total sample, no consistent type of preferred reinforcer was identified, with large variability evident. However, importantly, variability was significantly associated with the genetic subtype of AS: children without a deletion were more likely to be reinforced by social stimuli than children with a deletion. Most notably, all children with AS caused by an ICD were reinforced by social stimuli. Whilst no consistent reinforcer was found across children, the results suggest careful selection of reinforcers may be important; some children showed almost zero levels of target behaviors in some sensory and/or social interaction conditions, despite the broad descriptions of behavioral phenotype noting sensory seeking and a drive for social contact. The results from the study have important implications for future interventions with children with AS and indicate: (1) that genetic subtype may be an important consideration when developing guidelines or advice regarding behavior and (2) that the assumption of a strong drive for social contact in AS is not ubiquitous.

The first aim of the study was to examine the effect of social and sensory rewards on the rates of acquisition in children with AS. Overall, the frequency of target behaviors was significantly higher in the presence of both sensory and social stimuli than control conditions. This supports previous literature examining the use of reinforcement in teaching paradigms in children with a severe intellectual disability (Green et al., 1988; Fisher et al., 1992). Whilst these findings are not novel, replicating many examinations within the general intellectual disability literature, there are limited examinations of the use of reinforcement with children with AS, in which deficits in learning relative to level of intellectual disability are widely reported (Jiang et al., 1998, 2010; Summers and Szatmari, 2009; Heald et al., 2013).

The second aim of the study was to examine the comparative rates of target behaviors across specific manipulations of elements of social reinforcement. There was no significant association between any of the components of social interaction manipulated (eye contact, verbal interaction, physical contact and, laughing and smiling) and the frequency of rewarded target behaviors. This may suggest that whilst social interaction functions as a reinforcer in AS, this may not be specific to any one component of interaction. These findings contradict the research suggesting that eye contact may be an important component of social interaction in AS (see Mount et al., 2011). Whilst no consistent social reinforcer was identified across participants, the results indicated that *type* of social interaction was still important for individual children. For the children who were reinforced by social stimuli, the majority (60%) were reinforced by only one social interaction condition (d-statistic > 0.5), with only one child reinforced by all social interaction conditions. This has implications for future interventions conducted with children with AS, suggesting that the type of social interaction needs to be established in order for it to function as a reinforcer most effectively.

Interestingly, across the total sample there was no significant difference in the frequency of target behaviors between sensory and social rewards, either overall, or when comparing the conditions that showed the highest rates of target behaviors (high preference stimulus and full social interaction). This is striking considering the AS literature, which consistently reports a heightened motivation for adult interaction (Horsler and Oliver, 2006a; Mount et al., 2011) compared to the relative paucity of research describing sensory seeking behaviors. However, the analysis across genetic subtypes of AS (deletion and non-deletion) indicates a more nuanced profile of phenotypic behaviors within the AS population, with notable differences in behavior between the two genetic subtype groups. More specifically, a greater proportion of children without a deletion were reinforced by social stimuli. It is unlikely that this difference can be attributed to the significant difference in adaptive behavior across the two groups because there was no association between the frequency of target behavior across sensory and social conditions and the level of children’s adaptive behavior, indicating that adaptive behavior alone cannot account for differences across the two groups.

Out of the seven children who did not have a deletion, five children had ICD. All children with ICD were reinforced by social stimuli, compared to 28.6% of children with a deletion. Whilst the sample size of children with ICD is very small, the results across the two groups are compelling. The plots of target behaviors in the social reinforcement assessment confirm the difference in behavior between the two groups. These results extend the growing literature on genotype-phenotype correlations within AS, which to date has primarily focused on adaptive behavior and cognition (see Gentile et al., 2010). The precise reason for behavioral differences is unclear. The striking differences in social behavior in the current study provides a convincing rationale for the further delineation of behavioral phenotypes across genetic subtypes and highlights the need to make this distinction within the literature. Additionally, these results indicate that the study of social behavior in children with AS caused by ICD might be optimal with regard to exploring kinship theory and that the learning paradigm employed can generate objective metrics of motivation for social contact.

The results from the current study extend the research on kinship theory, suggesting that in addition to seeking social resources, some children with AS clearly find adult social interaction extremely rewarding and enjoyable. However, the variability in reinforcer efficacy across participants is striking. Overall, social stimuli functioned as a reinforcer for only 50% of all participants. Whilst the genetic subtype of AS partially accounted for this variability, this alone does not explain the marked behavioral differences across children and the contrasting findings to the broader literature on sociability in AS. One important factor, which relates to kinship theory, may be the familiarity of the researcher. Although time was spent interacting with children before beginning the assessments, the researcher remained “unfamiliar” compared to a parent or caregiver. It could be argued that based on the predictions from kinship theory, heightened behaviors relating to securing maternal resources are displayed, suggesting that the familiarity of the adult may be an important factor in the effectiveness of social interaction as a reinforcer. This is supported by observations of social behavior across familiar and unfamiliar adults in AS, with a higher frequency of social approach behavior toward a caregiver in the presence of certain conditions of social interaction (Mount et al., 2011). Once again, this highlights the need to further delineate the social phenotype of AS, describing the specific environmental conditions under which heightened sociability is evidenced.

There are some limitations to the study which may affect the reliability and validity of the findings. The main limitation of the study is the small number of participants across each genetic subtype. As only seven children were recruited who did not have a deletion, a more fine grained analysis of the data could not be conducted. Five participants in the non-deletion group had ICD. As a consequence, ICD accounted for the majority of the non-deletion sample (5/7), but due to the small numbers could not be treated as a separate participant group. The small numbers of participants across each genetic subtype reflects the relative rarity of AS not caused by a *de novo* deletion; whilst the prevalence of AS is 1 in 10,000, only 2% of cases are caused by ICD. However, the behavioral differences across groups were statistically significant even with small numbers of participants, suggesting that the sample size did not impact significantly on the findings from the study.

A second limitation is the short condition duration (2 min) for both reinforcement and preference assessments in comparison to previous research employing a similar methodology (Hagopian et al., 2004). During pilot work establishing the efficacy of the proposed methodology, participants exhibited high levels of aggressive behavior and distress during the preference assessments and reinforcer assessment control conditions. Interestingly, this observation occurred more often in conditions where social interaction was withheld. This is consistent with previous literature reporting aggression and social motivation in AS (Strachan et al., 2009; Allen et al., 2010; Arron et al., 2011). As a consequence, condition durations were limited to 2 min. However, although it could be argued that the short duration of conditions may account for the lack of distinction across social reinforcement conditions, this argument is negated by the striking difference in target behaviors observed across the low preference and high preference stimuli.

Overall, the results suggest that sensory and social stimuli can function as highly effective reinforcers in AS. Whilst no consistent reinforcer was identified across the group, the study highlights the importance of bespoke rewards for children in order to increase behavior more rapidly or prevent inadvertent reward of behaviors such as aggression. This has important implications for future interventions within this population, particularly given the slow rate of acquisition consistently reported in the literature and the high prevalence of aggression. Variability in reinforcer efficacy was partially accounted for by genetic subtype, with a greater proportion of children without a deletion reinforced by social interaction. This may have important implications for early intervention, given the literature suggesting an association between social motivation and aggression in AS (Strachan et al., 2009; Allen et al., 2010). Overall, the results have important implications for learning in AS and further study of kinship theory and highlight the need to further delineate genotype-phenotype correlations.

## Data Availability Statement

The raw data supporting the conclusions of this article will be made available by the authors, without undue reservation.

## Ethics Statement

The studies involving human participants were reviewed and approved by the University of Birmingham under application ERN_12-0018P. Written informed consent to participate in this study was provided by the participants’ legal guardian/next of kin.

## Author Contributions

MH and CO designed the study, oversaw the data collection and analysis, and drafted the manuscript. DA oversaw the data collection and analysis, and commented on drafted manuscript. EW collected the data. All authors contributed to the article and approved the submitted version.

## Conflict of Interest

The authors declare that the research was conducted in the absence of any commercial or financial relationships that could be construed as a potential conflict of interest.

## Footnotes

1 Although 22 children participants were recruited, the data from one participant were excluded as they became distressed during the assessments.

2 Information about the size of deletion (Class I or II) was not available for all participants.

## References

[B1] Allen D. Oliver C. Webster P. Reid D. Villa D. Beaumont S. (2010). Behavioural intervention for challenging behaviour in children with Angelman syndrome. *J. Intel. Disab. Res.* 54 885–885.

[B2] Arron K. Oliver C. Moss J. Berg K. Burbidge C. (2011). The prevalence and phenomenology of self-injurious and aggressive behaviour in genetic syndromes. *J. Intel. Disab. Res.* 55 109–120. 10.1111/j.1365-2788.2010.01337.x 20977515

[B3] Berument S. K. Rutter M. Lord C. Pickles A. Bailey A. (1999). Autism screening questionnaire: diagnostic validity. *Br. J. Psychiatry.* 175 444–451. 10.1192/bjp.175.5.444 10789276

[B4] Boyd S. Harden A. Patton M. (1988). The EEG in early diagnosis of the Angelman (happy puppet) syndrome. *Eur. J. Pediatr.* 147 508–513. 10.1007/bf00441976 3409926

[B5] Brown W. M. Consedine N. S. (2004). Just how happy is the happy puppet? An emotion signaling and kinship theory perspective on the behavioral phenotype of children with Angelman syndrome. *Med. Hypoth.* 63 377–385. 10.1016/j.mehy.2004.05.010 15288352

[B6] Buckley R. H. Dinno N. Weber P. (1998). Angelman syndrome: are the estimates too low? *Am. J. Med. Genet.* 80 385–390. 10.1002/(sici)1096-8628(19981204)80:4<385::aid-ajmg15>3.0.co;2-9 9856568

[B7] Bürger J. Buiting K. Dittrich B. Groß S. Lich C. Sperling K. (1997). Different mechanisms and recurrence risks of imprinting defects in Angelman syndrome. *Am. J. Hum. Genet.* 61 88–93. 10.1086/513900 9245988 PMC1715864

[B8] Calculator S. N. (2002). Use of enhanced natural gestures to foster interactions between children with Angelman syndrome and their parents. *Am. J. Speech Lang. Pathol.* 11:340. 10.1044/1058-0360(2002/039)

[B9] Calculator S. N. Black T. (2010). Parents’ priorities for AAC and related instruction for their children with Angelman Syndrome. *Augment. Alter. Commun.* 26 30–40. 10.3109/07434610903585406 20196702

[B10] Clayton-Smith J. Laan L. (2003). Angelman syndrome: a review of the clinical and genetic aspects. *J. Med. Genet.* 40 87–95. 10.1136/jmg.40.2.87 12566516 PMC1735357

[B11] Cliff N. (1993). Dominance statistic- ordinal analyses to answer ordinal questions. *Psychol. Bull.* 114 494–509. 10.1037/0033-2909.114.3.494

[B12] Dan B. Chéron G. (2004). Postural rhythmic muscle bursting activity in Angelman syndrome. *Brain Dev.* 26 389–393. 10.1016/j.braindev.2003.12.002 15275702

[B13] Didden R. Korzilius H. Smits M. G. Curfs L. M. (2004). Sleep problems in individuals with Angelman syndrome. *J. Inform.* 109 275–284. 10.1352/0895-8017(2004)109<275:spiiws>2.0.co;2 15176919

[B14] Engel E. (1993). Uniparental disomy revisited: the first twelve years. *Am. J. Med. Genet.* 46 670–674. 10.1002/ajmg.1320460613 8362910

[B15] Fisher W. Piazza C. C. Bowman L. G. Hagopian L. P. Owens J. C. Slevin I. (1992). A comparison of two approaches for identifying reinforcers for persons with severe and profound disabilities. *J. Appl. Behav. Anal.* 25 491–498. 10.1901/jaba.1992.25-491 1634435 PMC1279726

[B16] Gentile J. K. Tan W.-H. Horowitz L. T. Bacino C. A. Skinner S. A. Barbieri-Welge R. (2010). A neurodevelopmental survey of Angelman syndrome with genotype-phenotype correlations. *J. Dev. Behav. Pediatr.* 31:592. 10.1097/DBP.0b013e3181ee408e PMC2997715 20729760

[B17] Green C. W. Reid D. H. White L. K. Halford R. C. Brittain D. P. Gardner S. M. (1988). Identifying reinforcers for persons with profound handicaps: staff opinion versus systematic assessment of preferences. *J. Appl. Behav. Anal.* 21 31–43. 10.1901/jaba.1988.21-31 2967274 PMC1286091

[B18] Hagopian L. P. Long E. S. Rush K. S. (2004). Preference assessment procedures for individuals with developmental disabilities. *Behav. Modif.* 28 668–677. 10.1177/0145445503259836 15296524

[B19] Hagopian L. P. Rush K. S. Lewin A. B. Long E. S. (2001). Evaluating the predictive validity of a single stimulus engagement preference assessment. *J. Appl. Behav. Anal.* 34 475–485. 10.1901/jaba.2001.34-475 11800186 PMC1284341

[B20] Haig D. Wharton R. (2003). Prader−Willi syndrome and the evolution of human childhood. *Am. J. Hum. Biol.* 15 320–329. 12704708 10.1002/ajhb.10150

[B21] Heald M. Adams D. Oliver C. (2020). Profiles of atypical sensory processing in Angelman, cornelia de lange and fragile X syndromes. *J. Intel. Disab. Res.* 64 117–130. 10.1111/jir.12702 31828905

[B22] Heald M. Allen D. Villa D. Oliver C. (2013). Discrimination training reduces high rate social approach behaviors in Angelman syndrome: proof of principle. *Res. Dev. Disabil.* 34 1794–1803. 10.1016/j.ridd.2013.02.012 23518390

[B23] Horsler K. Oliver C. (2006a). The behavioural phenotype of Angelman syndrome. *J. Intel. Disab. Res.* 50 33–53. 10.1111/j.1365-2788.2005.00730.x 16316429

[B24] Horsler K. Oliver C. (2006b). Environmental influences on the behavioral phenotype of Angelman syndrome. *Am. J. Ment. Retard.* 111:311. 10.1352/0895-8017(2006)111[311:eiotbp]2.0.co;2 16968140

[B25] Hyman P. Oliver C. Hall S. (2002). Self-injurious behavior, self-restraint, and compulsive behaviors in Cornelia de Lange syndrome. *J. Inform.* 107 146–154. 10.1352/0895-8017(2002)107<0146:sibsra>2.0.co;2 11853532

[B26] Jiang Y.-h Armstrong D. Albrecht U. Atkins C. M. Noebels J. L. Eichele G. (1998). Mutation of the Angelman ubiquitin ligase in mice causes increased cytoplasmic p53 and deficits of contextual learning and long-term potentiation. *Neuron* 21 799–811. 10.1016/s0896-6273(00)80596-6 9808466

[B27] Jiang Y.-h. Pan Y. Zhu L. Landa L. Yoo J. Spencer C. (2010). Altered ultrasonic vocalization and impaired learning and memory in Angelman syndrome mouse model with a large maternal deletion from Ube3a to Gabrb3. *PLoS One* 5:e12278. 10.1371/journal.pone.0012278 20808828 PMC2924885

[B28] Jolleff N. Ryan M. (1993). Communication development in Angelman’s syndrome. *Arch. Dis. Childhood* 69 148–150. 8024300 10.1136/adc.69.1.148 PMC1029433

[B29] Keen D. Pennell D. (2010). Evaluating an engagement-based preference assessment for children with Autism. *Res. Aut. Spectr. Disord.* 4 645–652. 10.1016/j.rasd.2009.12.010

[B30] Kishino T. Lalande M. Wagstaff J. (1997). UBE3A/E6-AP mutations cause Angelman syndrome. *Nat. Genet.* 15 70–73. 10.1038/ng0197-70 8988171

[B31] Knoll J. Nicholls R. Magenis R. Graham J. Lalande M. Latt S. (1989). Angelman and Prader−Willi syndromes share a common chromosome 15 deletion but differ in parental origin of the deletion. *Am. J. Med. Genet.* 32 285–290. 10.1002/ajmg.1320320235 2564739

[B32] Kushlick A. Blunden R. Cox G. (1973). A method of rating behaviour characteristies for use in large scale surveys of mental handicap. *Psychol. Med.* 3 466–478. 10.1017/s0033291700054271 4762223

[B33] Mertz L. G. B. Thaulov P. Trillingsgaard A. Christensen R. Vogel I. Hertz J. M. (2014). Neurodevelopmental outcome in Angelman syndrome: genotype-phenotype correlations. *Res. Dev. Disabil.* 35 1742–1747. 10.1016/j.ridd.2014.02.018 24656292

[B34] Moncla A. Malzac P. Voelckel M.-A. Auquier P. Girardot L. Mattei M.-G. (1999). Phenotype-genotype correlation in 20 deletion and 20 non-deletion Angelman syndrome patients. *Eur. J. Hum. Genet.* 7:131. 10.1038/sj.ejhg.5200258 10196695

[B35] Mount R. Oliver C. Berg K. Horsler K. (2011). Effects of adult familiarity on social behaviours in Angelman syndrome. *J. Intel. Disab. Res.* 55 339–350. 10.1111/j.1365-2788.2010.01364.x 21255175

[B36] Oliver C. Berg K. Moss J. Arron K. Burbidge C. (2011). Delineation of behavioral phenotypes in genetic syndromes: characteristics of autism spectrum disorder, affect and hyperactivity. *J. Aut. Dev. Disord.* 41 1019–1032. 10.1007/s10803-010-1125-5 21080217

[B37] Oliver C. Horsler K. Berg K. Bellamy G. Dick K. Griffiths E. (2007). Genomic imprinting and the expression of affect in Angelman syndrome: what’s in the smile? *J. Child Psychol. Psychiatry* 48 571–579. 10.1111/j.1469-7610.2007.01736.x 17537073

[B38] Palmer J. Jenkins J. (1982). The ‘Wessex’ behavior rating system for mentally handicapped people: Reliability study. *Br. J. Ment. Subnorm*. 28 88–96. 10.1179/bjms.1982.011

[B39] Pearson E. Wilde L. Heald M. Royston R. Oliver C. (2019). Communication in Angelman syndrome: a scoping review. *Dev. Med. Child Neurol.* 61 1266–1274. 10.1111/dmcn.14257 31074506

[B40] Pelc K. Cheron G. Dan B. (2008). Behavior and neuropsychiatric manifestations in Angelman syndrome. *Neuropsych. Dis. Treat.* 4:577. 10.2147/ndt.s2749 18830393 PMC2526368

[B41] Penner K. A. Johnston J. Faircloth B. H. Irish P. Williams C. A. (1993). Communication, cognition, and social interaction in the Angelman syndrome. *Am. J. Med. Genet.* 46 34–39. 10.1002/ajmg.1320460108 8494032

[B42] Peters S. U. Goddard−Finegold J. Beaudet A. L. Madduri N. Turcich M. Bacino C. A. (2004). Cognitive and adaptive behavior profiles of children with Angelman syndrome. *Am. J. Med. Genet. Part A* 128 110–113. 10.1002/ajmg.a.30065 15213998

[B43] Peters S. U. Horowitz L. Barbieri-Welge R. Taylor J. L. Hundley R. J. (2012). Longitudinal follow−up of autism spectrum features and sensory behaviors in Angelman syndrome by deletion class. *J. Child Psychol. Psychiatry* 53 152–159. 10.1111/j.1469-7610.2011.02455.x 21831244

[B44] Prasad C. Wagstaff J. (1997). Genotype and phenotype in Angelman syndrome caused by paternal UPD 15. *Am. J. Med. Genet.* 70 328–329. 10.1002/(sici)1096-8628(19970613)70:3<328::aid-ajmg21>3.0.co;2-m 9188675

[B45] Rutter M. Bailey A. Lord C. (2003). *The Social Communication Questionnaire: Manual.* Palo Alto, CA: Western Psychological Services.

[B46] Strachan R. Shaw R. Burrow C. Horsler K. Allen D. Oliver C. (2009). Experimental functional analysis of aggression in children with Angelman syndrome. *Res. Dev. Disab.* 30 1095–1106. 10.1016/j.ridd.2009.03.005 19361955

[B47] Summers J. (2012). Neurodevelopmental outcomes in children with Angelman syndrome after 1 year of behavioural intervention. *Dev. Neurorehabil.* 15 239–252. 10.3109/17518423.2012.676101 22646082

[B48] Summers J. Szatmari P. (2009). Using discrete trial instruction to teach children with Angelman syndrome. *Focus Aut. Other Dev. Disabil.* 24 216–226. 10.1177/1088357609334057

[B49] Walz N. C. Baranek G. T. (2006). Sensory processing patterns in persons with Angelman syndrome. *Am. J. Occupat. Ther.* 60 472–479. 10.5014/ajot.60.4.472 16915878

[B50] Walz N. C. Benson B. A. (2002). Behavioral phenotypes in children with down syndrome, prader-willi syndrome, or Angelman syndrome. *J. Dev. Phys. Disabil.* 14 307–321.

[B51] Williams C. A. Beaudet A. L. Clayton−Smith J. Knoll J. H. Kyllerman M. Laan L. A. (2006). Angelman syndrome 2005: updated consensus for diagnostic criteria. *Am. J. Med. Genet. Part A* 140 413–418. 10.1002/ajmg.a.31074 16470747

